# Revalorization of Cava Lees to Improve the Safety of Fermented Sausages

**DOI:** 10.3390/foods10081916

**Published:** 2021-08-18

**Authors:** Salvador Hernández-Macias, Núria Ferrer-Bustins, Oriol Comas-Basté, Anna Jofré, Mariluz Latorre-Moratalla, Sara Bover-Cid, María del Carmen Vidal-Carou

**Affiliations:** 1 Departament de Nutrició, Ciències de l’Alimentació i Gastronomia, Facultat de Farmàcia i Ciències de l’Alimentació, Campus de l’Alimentació de Torribera, Universitat de Barcelona (UB), Av. Prat de la Riba 171, 08921 Santa Coloma de Gramenet, Spain; salva.hernandez@ub.edu (S.H.-M.); oriolcomas@ub.edu (O.C.-B.); mariluzlatorre@ub.edu (M.L.-M.); 2 Institut de Recerca en Nutrició i Seguretat Alimentària (INSA·UB), Universitat de Barcelona (UB), Av. Prat de la Riba 171, 08921 Santa Coloma de Gramenet, Spain; 3 Xarxa d’Innovació Alimentària (XIA), C/Baldiri Reixac 4, 08028 Barcelona, Spain; 4 Food Safety and Functionality Programme, Institute of Agrifood Research and Technology (IRTA), Finca Camps i Armet s/n, 17121 Monells, Spain; nuria.ferrer@irta.cat (N.F.-B.); anna.jofre@irta.cat (A.J.); sara.bovercid@irta.cat (S.B.-C.)

**Keywords:** cava lees, phenolic extract, food by-product, lactic acid bacteria, fermented sausages, *Salmonella* spp., *Listeria monocytogenes*, revalorization

## Abstract

The revalorization of food processing by-products not only reduces the environmental impact of their disposal, but also generates added economic value. Cava lees consist of inactive cells of *Saccharomyces cerevisiae*, and though regarded as a valueless winery by-product, they are rich in fiber and phenolic compounds. In this study, a challenge test was performed to assess the effect of cava lees and a phenolic extract (LPE) derived therefrom on the behaviour of technological microbiota (lactic acid bacteria used as a starter culture) and the foodborne pathogens *Salmonella* spp. and *Listeria monocytogenes* during the fermentation and ripening of pork sausages. Ten batches of fermented sausages were prepared with and without cava lees or the LPE, and with or without different strains of *Latilactobacillus sakei* (CTC494 or BAP110). The addition of cava lees reduced the pH values of the meat batter throughout the fermentation and ripening process. No growth-promoting effect on spontaneous lactic acid bacteria (LAB) or the starter culture was observed. In contrast, the presence of cava lees prevented the growth of the tested pathogens (*Salmonella* and *L. monocytogenes*), as did the starter culture, resulting in significantly lower counts compared to the control batch. In addition, the combination of cava lees with *L. sakei* CTC494 had a bactericidal effect on *Salmonella*. LPE supplementation did not affect the pH values or LAB counts but reduced the mean counts of *Salmonella*, which were 0.71 log_10_ lower than the control values at the end of the ripening. The LPE did not exert any additional effect to that of the starters applied alone. The revalorization of cava lees as a natural ingredient to improve the microbiological safety of fermented sausages is a feasible strategy that would promote a circular economy and benefit the environment.

## 1. Introduction

The food industry generates large amounts of by-products, whose disposal is costly from both an economic and environmental point of view [1]. Nowadays, there is growing interest in the revalorization of by-products rich in components such as polyphenols, proteins, fiber or lipids, which may have technological, nutritional and food safety applications [2].

The use of fiber-rich by-products as natural ingredients is being widely evaluated as an innovative reformulation strategy of fermented foods to achieve positive nutritional effects, such as the reduction of fat and/or the increase of dietary fiber content [3,4,5,6,7,8]. From a technological perspective, plant-derived by-products have been used in fermented food manufacture to promote the growth of lactic acid bacteria (LAB) and thus accelerate the fermentation process, with promising preliminary results [8,9,10,11]. Another widely explored revalorization strategy has focused on upcycling phenolic compounds from plant by-products as natural antioxidants or antimicrobial compounds for the formulation of different food products [12,13,14,15,16,17,18,19]. It has been demonstrated, although mostly in vitro assays, that phenolic extracts from by-products, such as grape (seeds, skins and stems), olive and apple pomace, and shitake stems, have a protective effect against foodborne pathogens [18,20,21,22,23,24,25,26]. In fact, it has been verified that some phenolic extracts help reduce the growth of some of the most common foodborne pathogenic bacteria (i.e., *Salmonella* spp., *Escherichia coli*, *Staphylococcus aureus* and *Listeria monocytogenes*) [27,28,29,30]. However, the minimum inhibitory concentration against a specific pathogen can vary depending on factors such as the type of polyphenol or the bacterial strain [27].

Cava lees are a by-product of the second fermentation of cava (Spanish sparkling wine), with an estimated annual production of ca. 300 tons [31]. Cava lees consist of inactive and plasmolyzed cells of *Saccharomyces cerevisiae* and are naturally rich in fiber (β-glucans and mannan-oligosaccharides). Moreover, sustained contact with the wine during the aging process enriches cava lees with significant amounts of phenolic compounds and organic acids. Despite their interesting composition, and the fact that cava lees account for a high percentage of total winery by-products (ca. 25%), their revalorization in food applications has not been explored to date [32]. Our research group recently demonstrated that under in vitro laboratory conditions cava lees have a growth-promoting effect on specific strains of LAB species commonly used as probiotics and/or starter cultures [33]. 

Considering the richness of cava lees in different types of fiber, polyphenols and organic acids, they have potential for revalorization in food applications. A possible function is to improve the microbiological safety of fermented sausage, as cava lees can enhance the implantation and growth of fermentative LAB and have an antimicrobial effect against pathogenic bacteria. In this framework, the aim of the present study was to assess the effect of cava lees and a derived phenolic extract on technological microbiota (i.e., LAB) and the foodborne pathogens *Salmonella* spp. and *Listeria monocytogenes* during the fermentation and ripening of pork sausages using a challenge test. To the best of our knowledge, this is the first time that the use of cava lees and its phenolic extract has been studied with an application in food safety through a challenge test in a more complex food matrix.

## 2. Materials and Methods

### 2.1. Bacterial Strains

*Listeria monocytogenes* strains CTC1034 (serotype 4b), 12MOB045LM (genoserotype II) and Scott A (serotype 4b) and *Salmonella enterica* strains CTC1003 (serotype London), CTC1756 (serotype Derby) and CCUG34136 (serotype Enteritidis, Type strain) were used for the challenge test. Cultures were prepared by growing each strain independently in brain heart infusion (BHI, Beckton Dickinson, Sparks, MD, USA) at 37 °C for 7 h and subsequently sub-cultured again at the same temperature for 18 h to reach the stationary phase. The bioprotective *Latilactobacillus sakei* (formerly *Lactobacillus sakei*) CTC494, a meat isolate producing sakacin k [34], and *L. sakei* BAP110 grown at 30 °C for 19 h in MRS broth were used as starter cultures. All cultures were preserved frozen at −80 °C in the growth medium supplemented with 20% glycerol as the cryoprotectant until used.

### 2.2. Preparation of Cava Lees

Cava lees were provided by the winery Freixenet S.A. (Sant Sadurní d’Anoia, Spain). The characterization of the composition of cava lees is detailed by Aguilera-Curiel [35] and they are mainly composed of polysaccharides (72.3% in wet weight) and a lesser percentage of proteins (8.5% in wet weight). Wet lees were centrifuged at 18,000× *g* for 10 min to remove the remaining cava. The lees were subsequently frozen in an ultra-low temperature freezer (−80 °C), freeze-dried (Cryodos-50, Telstar, Terrassa, Spain) and ground. Lyophilized lees (pH = 3.2 ± 0.02) were preserved in sealed tubes protected from the light and humidity.

### 2.3. Phenolic Extract Preparation

The cava lees phenolic extract (LPE) was obtained according to the method described by Silva et al. [24] with some modifications. Thus, 1 g of powder lees was added to 10 mL of a mixture of ethanol/water/acetic acid (80/20/0.05) and sonicated for 30 min. The supernatant was isolated by centrifugation (2500× *g* for 10 min at 4 °C), transferred into a flask, and the pellet was re-extracted. The collected supernatants were evaporated under vacuum on a rotatory evaporator at 37 °C. The dry residue was weighted and stored at 4 °C until used in the sausage elaboration. The total phenolic content of the extract, expressed as mg of gallic acid equivalents (GAE)/g, was determined using the method described by Vallverdú-Queralt et al. [36].

### 2.4. Elaboration of Inoculated Fermented Pork Sausages

Meat batter was prepared on a pilot scale under biosafety conditions by mixing minced lean pork meat and fat (8:2) ground through a 6 mm plate and inoculating it with a mixture of the three *L. monocytogenes* and three *Salmonella* strains (same amount for each strain) at a level of ca. 6 log_10_ CFU/g. Subsequently, the ground meat was mixed with (in g/kg) sodium chloride (25), dextrose (7), black pepper (3), sodium ascorbate (0.5), sodium nitrite (0.15) and potassium nitrate (0.15). In the corresponding batches, 5% (*w*/*w*) of cava lees or 0.3% (*w*/*w*) of LPE was also added (corresponding to the content of phenolic compounds expected in 5% of lees). According to a previous study, 5% of cava lees was the most effective concentration for enhancing the in vitro bacterial growth [33]. In addition, this percentage of lees is also similar to those of others plant-based by-products used in some other studies [4,5,6,10]. The total amount of water of the sausage recipe was 2.6 mL/kg, including the volume used as a vehicle to add the pathogen mixture to the ground meat and the starter culture if required. In total, 10 batches were prepared for the two experiments (Table 1).

For each batch, 80 g portions of the prepared meat batter were stuffed into Tublin10 (Tub-Ex, Tass, Denmark) permeable plastic bags using vacuum packaging and were submitted to a process of fermentation and drying consisting of 2 days at 23 °C and subsequently 19 days at 15 °C.

### 2.5. Microbiological and Physicochemical Analysis

For the microbiological analysis, ca. 15 g of sausage was diluted 10-fold in saline solution (0.85% NaCl and 0.1% Bacto Peptone (Beckton Dickinson, Franklin Lakes, NJ, USA), homogenized in a Blender Smasher^®^ bag (bioMérieux, Marcy-l’Etoile, France) for 1 min and again 10-fold serially diluted in saline solution. *L. monocytogenes* was enumerated on the chromogenic agar CHROMagar Listeria (CHROMagar, Paris, France) after incubation at 37 °C for 48 h. *Salmonella* was enumerated on the chromogenic agar CHROMagar Salmonella Plus (CHROMagar, Paris, France) after incubation at 37 °C for 24 h. LAB were enumerated in MRS (de Man, Rogosa and Sharpe; Merck, Darmstadt, Germany) agar plates incubated at 30 °C for 72 h under anaerobiosis using sealed jars with an AnaeroGen sachet (Oxoid Ltd., Altrincham, UK).

The pH was measured with a puncture electrode 5232 (Crison Instruments S.A., Alella, Spain) and a portable pHmeter PH25 (Crison Instruments S.A., Alella, Spain) and a_w_ with an Aqualab 3TE device (Decagon Devices, Inc. Pullman, WA, USA) at 25 °C. Analysis was performed in duplicate at selected sampling times throughout the fermentation and ripening process. 

### 2.6. Isolation and Monitoring of Starter Culture Strains

To monitor the implantation of the starter cultures, eight colonies per batch were isolated from MRS plates at day 0, 8 or 9 and 21 and submitted to Repetitive Extragenic Palindromic(REP)-PCR and Enterobacteria Repetitive Intergenic Consensus (ERIC)-PCR with primers FW-REP1R-I (5′-IIIICGICGICATCIGGC-3′) and RV-REP2-I1 (5′-ICGICTTATCIGGCCTAC-3′), and FW-ERIC R1 (5′-ATGTAAGCTCCTGGGGATTCAC-3′) and RV-ERIC 2 (5′-AAGTAAGTGACTGGGGTGAGCG-3′), respectively, under the conditions described in Rubio et al. [37].

### 2.7. Statistical Analysis

Analysis of variance (ANOVA) and the post-hoc Tukey HSD test at a *p* < 0.05 significance level was done using JMP software (SAS Institute Inc, Cary, NC, USA). To determine statistical differences in bacterial counts, pH and a_w_ of each batch during the manufacturing or storage period, one-way ANOVA was performed, using “Time” as a fixed factor.

## 3. Results and Discussion

### 3.1. Effect of Cava Lees Applied in Fermented Pork Sausages (Experiment 1)

Firstly, a challenge test with *Salmonella* spp. and *L. monocytogenes* was carried out in fermented sausages spontaneously fermented or inoculated with the starter culture *L. sakei* CTC494, both with and without the addition of 5% of lyophilized cava lees. 

#### 3.1.1. Characterization of Physicochemical Parameters

**Figure 1:**
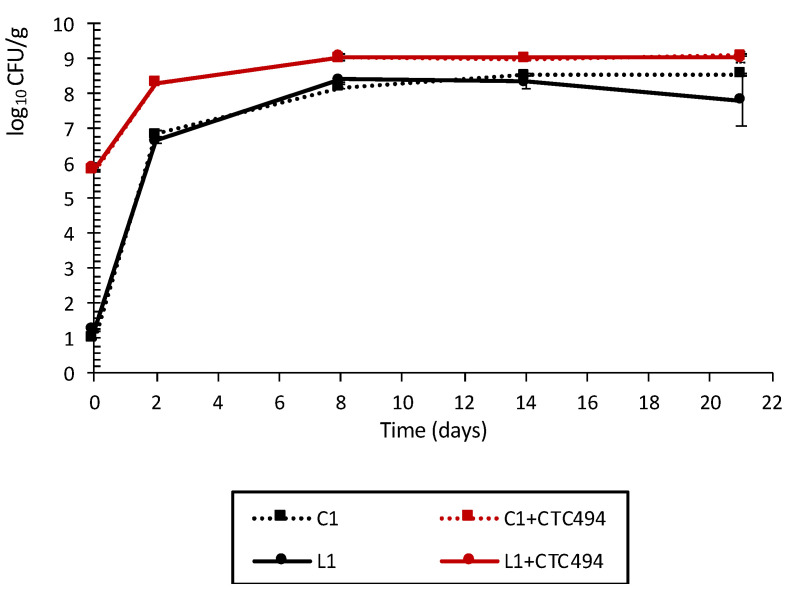
Growth of LAB in spontaneously fermented pork sausages with (L1) or without (C1) the addition of 5% of cava lees or fermented with the starter culture *L. sakei* CTC494, with (L1 + CTC494) or without (C1 + CTC494) cava lees.

Sausages supplemented with 5% (*w*/*w*) of cava lees initially had lower pH values than the unsupplemented batches due to the acidity of this winery by-product (pH 3.2 ± 0.02) (Table 2). During fermentation, the pH of sausages inoculated with the starter culture dropped significantly to values <5.3 (*p* < 0.05), while spontaneously fermented sausages underwent slower and slighter acidification (L1 and C1), due to the initial low levels of LAB (Figure 1). The subsequent increase in pH values in all batches throughout the ripening process could be explained by the formation of alkaline compounds during proteolysis [38]. In all cases, the presence of lees was associated with lower pH values. The difference in pH units in spontaneously fermented sausages with and without lees (L1 and C1, respectively) ranged from 0.46 at time zero to 0.85 at the end of the ripening. Studies on the use of citrus by-products in fermented sausages also report lower pH values due to their intrinsic acidity (e.g., orange fiber by-product pH = 3.28) [8,10,39]. A synergic effect was observed when cava lees were combined with *L. sakei* CTC494 (L1 + CTC494), resulting in a final pH value 1.23 units lower than in the control (C1, without cava lees or starter culture). 

Values of a_w_ gradually decreased over the 21 days of ripening (Table 2) due to the sausage drying process, with no significant differences among batches (*p* > 0.05), neither by the inoculation of the starter culture nor by the addition of cava lees.

#### 3.1.2. Behavior of LAB during Fermentation and Ripening

Figure 1 shows the growth of LAB in the different batches of sausages during fermentation and ripening. The batches formulated with a starter culture (C1 + CTC494 and L1 + CTC494) exhibited the highest LAB counts throughout the process, ranging from the initial inoculated level of 5.9 log_10_ CFU/g to more than 9 log_10_ CFU/g from day 8, and remaining stable thereafter. The implantation of the *L. sakei* CTC494 starter culture was confirmed by RAPD-PCR, with 100% (eight out of eight) of the isolated colonies showing the same RAPD profile as the starter culture strain at the end of the ripening process. In contrast, LAB levels in sausages produced without a starter culture (C1 and L1) were initially ca. 1.2 log_10_ CFU/g and reached 8.3 log_10_ CFU/g at day 8. During the subsequent ripening process, the levels remained slightly lower than in sausages with a starter culture.

With the current study design and matrix composition, the addition of cava lees did not promote LAB growth compared to the control batches throughout the manufacturing process, whether using spontaneous fermentation or *L. sakei* CTC494. These results are not in accordance with those previously obtained in vitro, also using *L. sakei* CTC494. In that study, the supplementation of the culture medium with the same amount of cava lees (5%) resulted in a significantly higher concentration of cells in different LAB strains compared to the control (without lees); in the case of *L. sakei* CTC494, the maximum population density was 0.8 log_10_ units higher [33]. The lower amount of readily fermentable substrate in the fermented sausage formulation (i.e., 0.7% dextrose) compared to the in vitro culture media (i.e., MRS broth with 2% dextrose [33]) did not favor the use of cava lees fiber by LAB to promote their growth. A significant growth-promoting effect of other fiber-rich by-products on specific LAB strains has been demonstrated in laboratory media [40,41], whereas the addition of various by-products (from lemon, orange, tiger nut, peach or apple) in fermented sausages that also contained easily fermentable carbohydrates (e.g., glucose, dextrose, sucrose, lactose) did not improve LAB growth [3,5,8,42]. On the other hand, in the study of Yalınkılıç et al. [10], higher LAB counts were obtained in fermented sausages with 4% orange fiber compared to the control (without added fiber), although the difference in mean counts was low (0.24 log_10_ units).

#### 3.1.3. Impact of Cava Lees on Pathogenic Bacteria 

**Figure 2:**
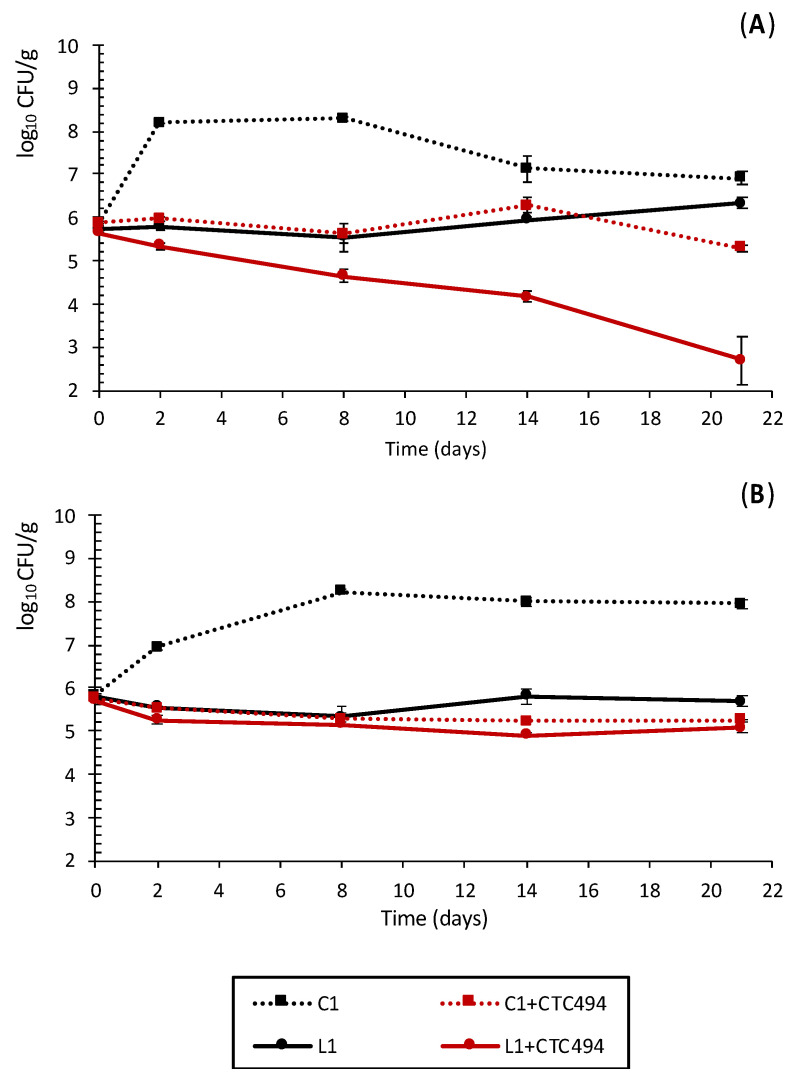
Counts of *Salmonella* (**A**) and *Listeria monocytogenes* (**B**) strains in pork sausages spontaneously fermented with (L1) and without (C1) the addition of 5% of cava lees or fermented with the starter culture *L. sakei* CTC494 with (L1+ CTC494) or without (C1+ CTC494) lees.

Figure 2 shows the behavior of the pathogenic bacteria during the fermentation and ripening of the four batches of pork sausages. The sakacin k-producing strain *L. sakei* CTC494 was selected as a bioprotective culture able to inhibit the growth of *L. monocytogenes* [34]. Although sakacin has no specific inhibitory effect on Gram-negative bacteria such as *Salmonella*, the presence of the starter culture accelerated acidification and resulted in a lower pH, which is known to enhance the inactivation of *Salmonella* [43]. The presence of 5% of cava lees also had an anti-pathogenic effect, reducing the load of *Salmonella* and *L. monocytogenes* in both types of fermented sausages (Figure 2).

Regarding the antimicrobial (growth inhibition) effect against *Salmonella*, significantly lower counts were recorded in sausages formulated with lees (L1) at all sampling times, being up to 2.7 log_10_ and 0.6 log_10_ lower than in C1 (*p* < 0.05) at day 8 and 21, respectively. It is important to highlight that the effect of cava lees on the *Salmonella* levels was similar to that exerted by the starter culture. Moreover, combining cava lees with *L. sakei* CTC494 (L1 + CTC494) enhanced the antimicrobial effect, resulting in a reduction of 3 log_10_ in *Salmonella* during the fermentation and ripening, which was due to both bacteriostatic and strong bactericidal effects. At the end of the process (day 21), *Salmonella* counts were 4.3 log_10_ lower than in control sausages (C1, *p* < 0.05).

The growth inhibitory effect of cava lees against *L. monocytogenes* was similar to that of bacteriocin-producing *L. sakei* CTC494. Compared to the control, *L. monocytogenes* counts were 2.3 log_10_ and 2.9 log_10_ lower in fermented sausages formulated with cava lees applied alone or together with *L. sakei* CTC494, respectively (*p* < 0.05). 

To date, few studies have focused on the revalorization of by-products with antimicrobial effects against food-borne bacteria, especially in fermented products. A recent study revealed that a celery by-product powder produced a significant decrease in total *Enterobacteriaceae* counts in cooked sausages [44]. An inhibitory effect against pathogenic and opportunistic bacteria of an apple by-product added to fermented milk permeate beverages has also been recently reported [45]. Conversely, in a study on fermented sausages supplemented with a lemon by-product, higher levels of *Listeria innocua* (used as a surrogate of *L. monocytogenes*) were recorded in comparison with the unsupplemented sausages [8]. In contrast, far more studies have assessed the antimicrobial effect of food by-product extracts rich in bioactive compounds such as polyphenols [14,18,19,24,46]. 

In the current study, besides the growth-inhibitory effect of the starter culture *L. sakei* CTC494, the lower pH values achieved at the beginning of fermentation (ca. 5.2 at day 0) in sausages supplemented with cava lees (L1 and L1 + CTC494) could be another major factor responsible for the lower pathogen counts in these batches. In fact, in hurdle technology for food preservation, the pH is considered a crucial hurdle in the control of pathogenic bacteria in fermented sausages, especially in combination with a lower a_w_ [47]. Compared to *Salmonella*, *L. monocytogenes* is more tolerant of the harsh environment usually found at the last stages of ripening, characterized by a low pH and a_w_ [43,48,49], which could explain its lower reduction in the batches formulated with cava lees. Finally, besides the effect of pH, components of cava lees such as polyphenols and/or organic acids could also play a role in the antimicrobial activity of this by-product. It is worth highlighting that polyphenols tend to be more active against Gram-positive than Gram-negative bacteria, which can be attributed to the different bacterial cell wall structures [19,24,27,29,50].

### 3.2. Effect of the LPE in Fermented Pork Sausages (Experiment 2)

In order to investigate whether the antimicrobial activity of cava lees could be also attributed to its phenolic fraction, a second challenge test with *Salmonella* and *L. monocytogenes* was carried out in fermented pork sausages formulated with LPE instead of cava lees. Additionally, the effect of a bacteriocinogenic (*L. sakei* CTC494) and a non-bacteriocinogenic (*L. sakei* BAP110) starter culture was evaluated. 

The total phenolic content of the LPE was 152.2 ± 3.5 mg GAE/g. According to the literature, the total phenolic content of cava or wine lees differs widely, even among studies using the same extraction and determination methodology, with mean values ranging from 26 to 254 mg GAE/g [35,51,52]. Jara-Palacios et al. describe that the phenolic content in wine lees depends on the grape variety and other factors related with the vinification process [52]. The main phenolic compounds found in cava lees are caftaric acid, catechin and epicatechin, which are also the most abundant phenols in sparkling wines [35]. It has been reported that yeast cell walls possess a high capacity to adsorb phenolic compounds from wine [53,54,55,56], the contents often being greater than in other types of winery by-products (e.g., grape seeds, stems and skin) [57]. 

#### 3.2.1. Characterization of Physicochemical Parameters and LAB Counts

Table 3 shows the pH and a_w_ values of the different batches of fermented sausages. Overall, supplementation with the LPE did not affect the acidity of the product at day 0. Batches inoculated with starter cultures underwent a faster acidification. The lowest pH values were reached at days 5 and 14 of ripening in sausages prepared with *L. sakei* CTC494 and *L. sakei* BAP110, respectively. A reducing effect on the pH was also observed when the starter culture was applied with the LPE. Acidification in non-inoculated fermented sausages (C2 and E2) was slower and weaker, the lowest pH value being recorded at the end of the ripening process (ca. 5.3), as usually occurs in spontaneously fermented sausages [43,58]. The rise in pH is due to proteolysis phenomena that take place at the end of the ripening period. pH values up to 6.5 have been reported in Spanish fermented sausages [59]. However, this process is highly variable and not very controllable, especially in spontaneous fermented sausages. In fact, in the current study, a great variability could be observed at this point (day 21) in batch C1 (6.26 ± 0.38) than in C2 (5.28 ± 0.17). The use of different meat raw materials in the preparation of experiments 1 and 2 could also explain these differences, even though the batches were prepared under the same conditions. No differences in a_w_ were observed between batches during the process, as the same drying conditions were applied in each case.

**Figure 3:**
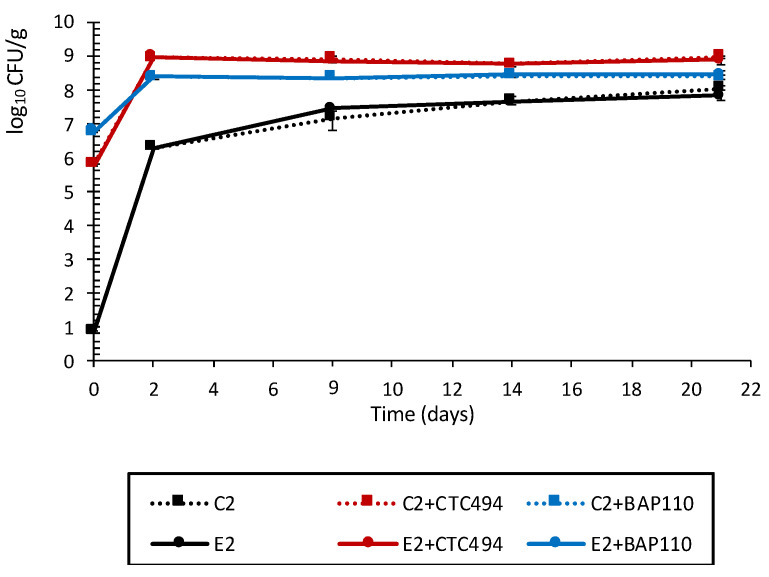
Growth of LAB in pork sausages spontaneously fermented with (E2) and without (C2) the addition of 0.3% LPE or fermented with the starter culture *L. sakei* CTC494 or *L. sakei* BAP110 and with (E2 + CTC494 or E2 + BAP110) or without (C2 + CTC494 or C2 + BAP110) the LPE.

As shown in Figure 3, batches with a fermentation process driven by all the strains of *L. sakei* starter culture (C2 + CTC494, E2 + CTC494, C2 + BAP110 and E2 + BAP110) achieved values of up to 9 log_10_ CFU/g of LAB at the first 2 days of ripening. These counts remained more or less stable until the end of the ripening process. The implantation of starter cultures was monitored by RAPD-PCR analysis, which showed that 100% (eight out of eight) of the isolates from the MRS plates at the end of the ripening had the same RAPD profile as the corresponding starter culture strain, thus confirming their competitiveness and dominance over the endogenous LAB. In batches without a starter culture, the initial levels of LAB were <1 log_10_ CFU/g, increasing up to 6.3 and 8 log_10_ CFU/g at 2 and 21 days, respectively. 

Overall, the addition of the LPE did not affect the LAB counts in any batch. In this context, LAB have been described as highly tolerant to polyphenols in the growth environment [50,60]. Our results agree with those of Wang et al. [61] and Zhang et al. [62], who found that LAB counts were unaltered by the addition of different polyphenols to meat products. Nevertheless, fermented sausages produced with a shiitake by-product extract had higher levels of LAB [16,17]. Ultimately, LAB tolerance of phenolic compounds, and their ability to metabolize them, seems to be strain- or species-specific [50,63].

#### 3.2.2. Impact of the LPE on Pathogenic Bacteria

**Figure 4:**
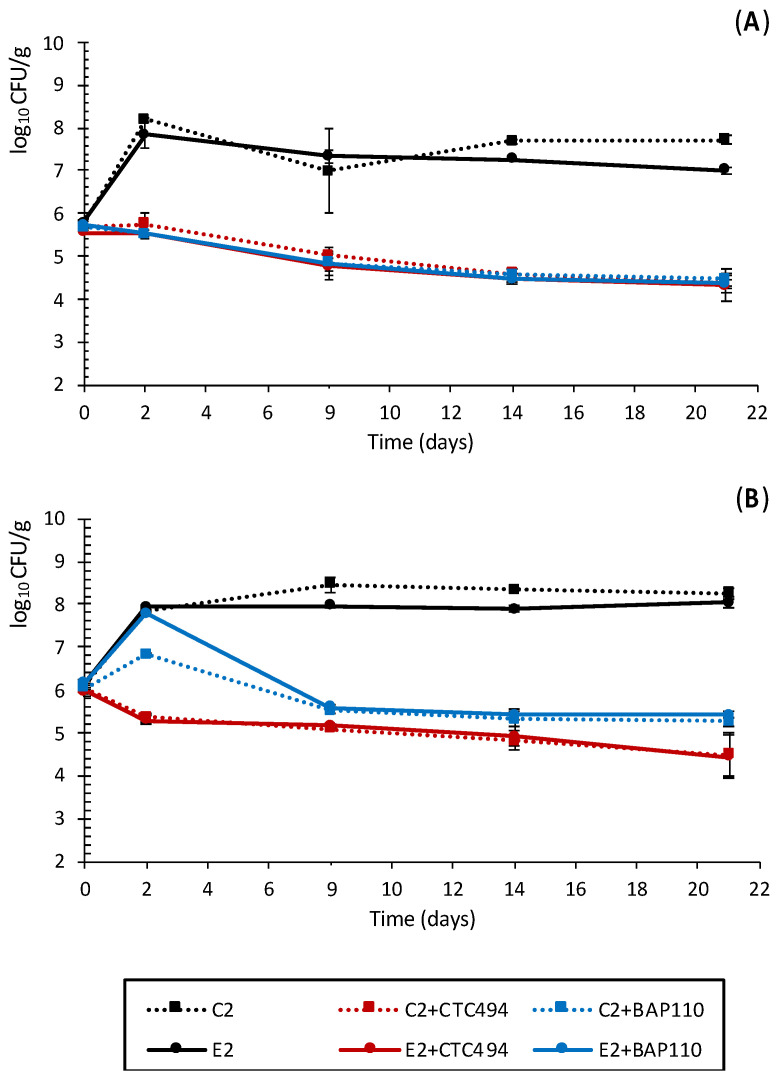
Behavior of *Salmonella* (**A**) and *Listeria monocytogenes* (**B**) strains in sausages spontaneously fermented with (E2) and without (C2) the addition of 0.3% of LPE or fermented with the starter culture *L. sakei* CTC494 or *L. sakei* BAP110 with (E2 + CTC949 or E2 + BAP110) or without (C2 + CTC494 or C2 + BAP110) the LPE.

The effect of the LPE, alone or combined with *L. sakei* CTC494 or *L. sakei* BAP110, against *Salmonella* and *L. monocytogenes* is shown in Figure 4. When no starter culture was added, the addition of the LPE had very little effect on *Salmonella*, whose growth during the first days of fermentation was similar to that of the control (without the LPE and starter culture). However, the mean counts of the pathogen at the end of the ripening were 0.71 log_10_ lower than in the control sausages. A similar effect was observed for *L. monocytogenes*, although in this case the final counts in the E2 batch were similar to the control sausages (C2).

As expected, *L. sakei*-based starter culture strains exerted a strong antimicrobial effect on *Salmonella* and *L. monocytogenes*, resulting in significantly lower pathogen counts (by 3–4 log_10_ units) at the end of ripening compared to the spontaneously fermented control sausages. No additional effect was observed when the LPE was added together with the starter cultures. No strain-specific effect was observed against *Salmonella*, which exhibited similar behavior with both starter cultures, in contrast with *L. monocytogenes*, whose behavior differed. The bacteriocinogenic strain *L. sakei* CTC494 not only prevented the growth of *L. monocytogenes* but exerted a listericidal effect from the early stages of fermentation and ripening. The non-bacteriocinogenic *L. sakei* BAP110 reduced but did not prevent the growth of *L. monocytogenes* during fermentation and had an inactivation effect during ripening; at the end of the process, the count was 1 log_10_ higher than in the batches containing the bacteriocinogenic *L. sakei* CTC494 (C2 + CTC494 and E3 + CTC494). This enhanced lethality can be related to the already reported specific antilisterial effect of *L. sakei* CTC494 in other food matrixes [34,64,65].

Polyphenols are widely reported to have an antimicrobial effect against several pathogenic bacteria, including *Staphylococcus aureus*, *Escherichia coli*, *Salmonella* spp. and *L. monocytogenes*, mostly in the context of microbial cultures [20,24,27,29,50,66]. The results obtained here indicate that the anti-*Salmonella* effect of whole cava lees in spontaneously fermented pork sausages may be partially attributed to the phenolic fraction of this by-product (Figure 4A). However, no effect of the LPE was observed against *L. monocytogenes*. Although most reports describe Gram-positive bacteria as far more susceptible to polyphenols than Gram-negative bacteria [20,24,28,67], this trend was not supported by the results of the current study.

The antimicrobial efficacy of polyphenol-rich extracts against pathogenic bacteria varies greatly, depending on both the phenolic structure and the bacterial species [27,63,67]. Cetin-Karaca et al. [29] assessed the antimicrobial potential of different plant phenolic compounds against three *Salmonella* species, reporting that (-)epicatechin, one of the main polyphenols found in cava lees, was the most effective, although with varying degrees of sensitivity according to the species. Conversely, among the range of bacterial species tested by Silva et al. [24], a winery by-product consisting of a grape polyphenol extract showed high antimicrobial activity against two food-borne strains of *L. monocytogenes*, but not *Salmonella*. It seems that the phenolic antimicrobial activity depends not only on the type of bacteria but also on the specific strain or serotype [27].

On the other hand, sausages fermented with *L. sakei* starter cultures exhibited a significant reduction in *Salmonella* and *L. monocytogenes* counts, regardless of the presence of the LPE. Similarly, Tremonte et al. [8] found that the addition of a polyphenol-rich lemon by-product did not enhance the anti-*Listeria* effect of a bioprotective strain of *Lactiplantibacillus plantarum* during the ripening of fermented sausages. Considering that LAB may be able to metabolize plant-derived polyphenols, thus significantly compromising their antimicrobial potential [50,60,63,68], it may be envisaged that in the current study, the endogenous LAB and the inoculated *L. sakei* technological strains could have reduced the polyphenol fraction of both the cava lees and LPE. 

Further studies could be designed to elucidate the role of specific components of cava lees in the antimicrobial effect observed in pork fermented sausages, which could at least be attributed to the lower pH and, most probably to acidic compounds (such as tartaric acid) and phenolic compounds (such as caftaric acid, catechin and epicatechin) determining the by-product acidity or other bacteriostatic properties. Furthermore, considering that sensory qualities are essential for consumer acceptance of food, the potential impact of cava lees on the sensorial profile of the final product should also be addressed.

## Figures and Tables

**Table 1:** Batches of fermented sausages formulated with or without cava lees or the lees phenolic extract (LPE) and/or different strains of *L. sakei* as the starter culture.

Experiment	Batch	Ingredient	Starter Culture
1	C1		
	L1	Cava lees	
	C1 + CTC494		*L. sakei* CTC494 ^1^
	L1 + CTC494	Cava lees	*L. sakei* CTC494 ^1^
2	C2		
	E2	LPE	
	C2 + CTC494		*L. sakei* CTC494
	E2 + CTC494	LPE	*L. sakei* CTC494
	C2 + BAP110		*L. sakei* BAP110
	E2 + BAP110	LPE	*L. sakei* BAP110

^1^ Producer of the bacteriocin sakacin K [34].

**Table 2:** Values of pH and a_w_ (mean ± standard deviation) during the fermentation and ripening of pork sausages. Batches included sausages formulated without (C1) or with (L1) cava lees, spontaneously fermented or with the addition of a starter culture (*L. sakei* CTC494).

		Batch			
	Day	C1	L1	C1 + CTC494	L1 + CTC494
pH	0	5.67 ± 0.01 ^a^	5.20 ± 0.14 ^b^	5.66 ± 0.02 ^a^	5.22 ± 0.09 ^b^
	2	5.74 ± 0.01 ^a^	5.43 ± 0.04 ^b^	5.07 ± 0.01 ^c^	4.89 ± 0.01 ^d^
	4	5.62 ± 0.02 ^a^	5.39 ± 0.01 ^b^	4.93 ± 0.01 ^c^	4.76 ± 0.01 ^d^
	8	5.44 ± 0.02 ^a^	5.35 ± 0.01 ^b^	5.01 ± 0.02 ^c^	4.80 ± 0.01 ^d^
	14	5.36 ± 0.05 ^a^	5.18 ± 0.03 ^ab^	5.40 ± 0.15 ^a^	4.91 ± 0.00 ^b^
	21	6.26 ± 0.38 ^a^	5.41 ± 0.05 ^b^	5.28 ± 0.05 ^b^	5.03 ± 0.05 ^b^
a_w_	0	0.973 ± 0.001 ^a^	0.972 ± 0.000 ^a^	0.973 ± 0.000 ^a^	0.972 ± 0.001 ^a^
	2	0.972 ± 0.000 ^a^	0.971 ± 0.000 ^a^	0.972 ± 0.000 ^a^	0.972 ± 0.000 ^a^
	4	0.974 ± 0.000 ^a^	0.972 ± 0.000 ^a^	0.969 ± 0.000 ^a^	0.970 ± 0.000 ^a^
	8	0.969 ± 0.000 ^a^	0.969 ± 0.000 ^a^	0.967 ± 0.000 ^b^	0.964 ± 0.000 ^c^
	14	0.963 ± 0.000 ^a^	0.964 ± 0.000 ^b^	0.965 ± 0.000 ^ab^	0.960 ± 0.000 ^c^
	21	0.960 ± 0.000 ^b^	0.966 ± 0.000 ^a^	0.962 ± 0.000 ^ab^	0.961 ± 0.000 ^b^

Values are mean ± standard deviation of triplicates. For each sampling day, significant differences between batches are indicated by different superscript letters (*p* < 0.05).

**Table 3:** Values of pH and a_w_ (mean ± standard deviation) during the fermentation and ripening of pork sausages. Batches included sausages elaborated without (C2) or with (E2) a cava lees phenolic extract, spontaneously fermented or with the addition of a starter culture (*L. sakei* CTC494 or *L. sakei* BAP110).

		Batch					
	Day	C2	E2	C2 + CTC494	E2 + CTC494	C2 + BAP110	E2 + BAP110
pH	0	5.88 ± 0.02 ^a^	5.86 ± 0.01 ^a^	5.88 ± 0.01 ^a^	5.86 ± 0.02 ^a^	5.85 ± 0.01 ^a^	5.77 ± 0.05 ^b^
	2	5.84 ± 0.01 ^a^	5.81 ± 0.02 ^a^	5.10 ± 0.03 ^c^	5.10 ± 0.03 ^c^	5.42 ± 0.10 ^b^	5.38 ± 0.11 ^b^
	5	5.71 ± 0.07 ^a^	5.73 ± 0.04 ^a^	4.96 ± 0.03 ^c^	4.95 ± 0.01 ^c^	5.24 ± 0.03 ^b^	5.33 ± 0.04 ^b^
	9	5.56 ± 0.07 ^a^	5.56 ± 0.02 ^a^	4.98 ± 0.01 ^b^	4.96 ± 0.01 ^b^	4.99 ± 0.02 ^b^	4.98 ± 0.02 ^b^
	14	5.38 ± 0.04 ^b^	5.46 ± 0.03 ^a^	5.01 ± 0.01 ^c^	5.00 ± 0.01 ^cd^	4.96 ± 0.02 ^cd^	4.95 ± 0.00 ^d^
	21	5.28 ± 0.17 ^a^	5.38 ± 0.09 ^a^	5.01 ± 0.01 ^b^	5.01 ± 0.04 ^b^	5.02 ± 0.02 ^b^	4.99 ± 0.02 ^b^
a_w_	0	0.972 ± 0.001 ^a^	0.971 ± 0.001 ^a^	0.972 ±0.001 ^a^	0.971 ± 0.001 ^a^	0.972 ± 0.001 ^a^	0.971 ± 0.001 ^a^
	2	0.970 ± 0.001 ^a^	0.970 ± 0.001 ^a^	0.972 ± 0.001 ^a^	0.971 ± 0.001 ^a^	0.971 ± 0.001 ^a^	0.971 ± 0.001 ^a^
	5	0.971 ± 0.002 ^a^	0.969 ± 0.002 ^a^	0.970 ± 0.001 ^a^	0.970 ± 0.001 ^a^	0.969 ± 0.001 ^a^	0.969 ± 0.001 ^a^
	9	0.972 ± 0.001 ^a^	0.971 ± 0.001 ^a^	0.971 ± 0.002 ^a^	0.972 ± 0.001 ^a^	0.969 ± 0.001 ^a^	0.970 ± 0.001 ^a^
	14	0.966 ± 0.001 ^bc^	0.970 ± 0.002 ^ab^	0.968 ± 0.001 ^abc^	0.971 ± 0.002 ^a^	0.964 ± 0.002 ^c^	0.969 ± 0.001 ^ab^
	21	0.964 ± 0.001 ^a^	0.963 ± 0.001 ^a^	0.963 ± 0.001 ^a^	0.963 ± 0.002 ^a^	0.962 ± 0.001 ^a^	0.963 ± 0.001 ^a^

Values are mean ± standard deviation of triplicates. For each sampling day, significant differences between batches are indicated by different superscript letters (*p* < 0.05).
